# The value of T_2_^*^-weighted gradient echo imaging for detection of familial cerebral cavernous malformation: A study of two families

**DOI:** 10.3892/etm.2012.845

**Published:** 2012-11-30

**Authors:** XUE-WU LIU, SHU-HUA WANG, ZHAO-FU CHI, LI-JUN SU, XIU-HE ZHAO, SHENG-JUN WANG

**Affiliations:** Department of Neurology, Qilu Hospital of Shandong University, Jinan, Shandong 250012, P.R. China

**Keywords:** diagnosis, familial cerebral cavernous malformation, gradient echo T_2_^*^-weighted imaging, magnetic resonance imaging

## Abstract

The aim of this study was to investigate the value of T_2_^*^-weighted gradient echo imaging (GRE T_2_^*^-WI) for the detection of familial cerebral cavernous malformation (FCCM). Twenty-six members of 2 families with FCCM were examined using computed tomography (CT), conventional magnetic resonance imaging (MRI) and GRE T_2_^*^-WI sequences. We identified 12 cases of FCCM using GRE T_2_^*^-WI sequences. These 12 patients had multiple lesions (mean 23). The lesions were most commonly located in the ganglia. Other areas included the cortex-subcortex, thalamus, cerebellum and brainstem. These lesions appeared as a reticulated core of mixed signal intensity with a surrounding rim of decreased signal intensity representing hemosiderin from previous hemorrhages. The mean numbers of lesions and cases of FCCM identified by various conventional MRI sequences were 5–17 and 3–9, respectively. Conventional MRI examination involved T_1_-weighted imaging (T_1_WI), T_2_-weighted imaging (T_2_WI), T_2_-fluid-attenuated inversion recovery (T_2_Flair), diffusion-weighted imaging (DWI) and spin-echo imaging (SE) sequences, in that order. The numbers of lesions identified by MRI were fewer than those identified by GRE T_2_^*^-WI. CT only identified 3 cases with large lesions combined with hemorrhage and calcification. These findings suggest that GRE T_2_^*^-WI is the first choice when diagnosing FCCM compared with CT and conventional MRI.

## Introduction

Computed tomography (CT) is an important method for the diagnosis of cerebral cavernous malformation (CCM) prior to the clinical application of magnetic resonance (MR). However, only large CCM lesions complicated with hemorrhage and calcification are visible in a CT scan. In addition, diagnoses of microangioma or CCM in the posterior cranial fossa and brainstem have been missed (1,2). Magnetic resonance imaging (MRI) has gradually improved the sensitivity of the diagnosis of CCM following the widespread use of MR, but certain sequences have conspicuous limitations. The definitive diagnosis rate of CCM almost equals that obtained from autopsy results when T_2_^*^-weighted gradient echo imaging (GRE T_2_^*^-WI) is used (3,4). GRE T_2_^*^-WI has been considered to be the most important diagnostic tool for CCM. Twelve patients, of which 2 were diagnosed with familial cerebral cavernous malformation (FCCM) from August, 2005 to June, 2007, were easily diagnosed with CCM by GRE T_2_^*^-WI following brain CT and conventional MRI examination.

## Materials and methods

### Patients

Twelve of 26 members in two families were diagnosed with FCCM (8 male, 4 female, aged 8 to 74 years, mean age 36.5). All patients were subjected to brain CT and conventional MR scans prior to GRE T_2_^*^-WI. Various symptoms were recorded, including repeated history of headache and dizziness (4 patients), hemiparalysis (4 patients), hemianesthesia (3 patients), speech disfluency (2 patients), hydroposia bucking and dysphagia (2 patients), seizure (1 patient), hemiablepsia (1 patient), skin angioma (6 patients) and asymptomatic manifestations (6 patients). Fourteen individuals presented with no clinical symptoms and no foci on MR scans. Each family member exhibited no vascular malformations upon examination of the fundus of the eye. All participants provided written informed consent before entering the study. The present study was approved by the ethics committee of Qilu Hospital of Shandong University (Jinan city, Shandong, China).

### MRI

All patients underwent conventional MRI using a 3.0T scanner (GE Signa EXCITE II; General Electric, Waukesha, WI, USA), including transverse and axial T_1_-weighted imaging (T_1_WI), T_2_-weighted imaging (T_2_WI), T_2_-fluid-attenuated inversion recovery (T_2_Flair), diffusion weighted imaging (DWI), magnetic resonance angiography (MRA) and spin-echo imaging (SE) sequences. GRE T_2_^*^-WI was carried out simultaneously [repeat time/echo time (TR/TE), 520/20 ms; flip angle 20°; section thickness, 6 mm; field of view (FOV), 24x18; matrix, 512x256]. The patients underwent a CT scan prior to MRI.

### MRI analysis

Two independent neuroradiologists evaluated the CT and MR images. First, they ascertained whether pathological changes were present in the brain. Second, they decided whether the changes were CCM and identified the different CCM foci shown on various MR sequences. Finally, they determined the location, size and quantity of vascular malformation based on the locations of CCM (cortex-subcortex, basal ganglia, thalamencephalon, brainstem and cerebellum).

### Statistical analysis

Data were expressed as the means ± standard deviation. One-way ANOVA and the Newman-Keuls test were used for statistical analysis of the results as appropriate. P<0.05 was considered to indicate a statistically significant result.

## Results

### Features of CCM in MRI and CT images

**Figure 1.**
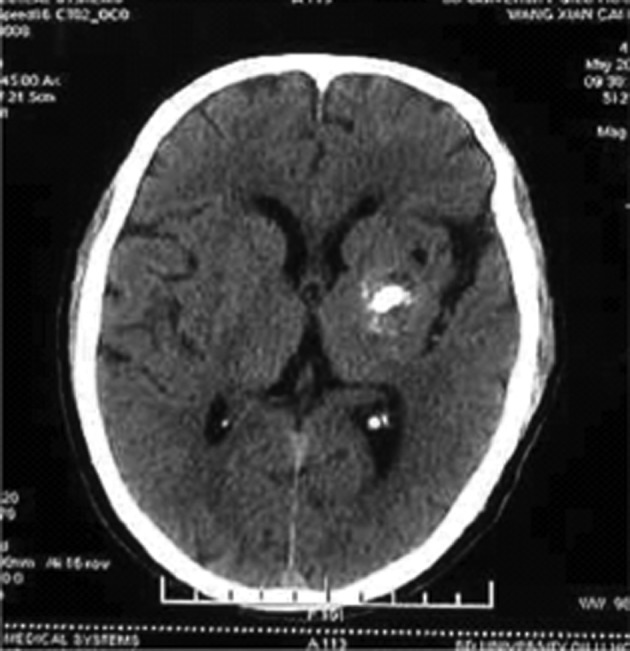
CCM on CT, the lesions complicated with hemorrhage and calcification are large enough to be visible on left basal ganglia. The foci show irregular mixed density. CCM, cerebral cavernous malformation; CT, computed tomography.

**Figure 2.**
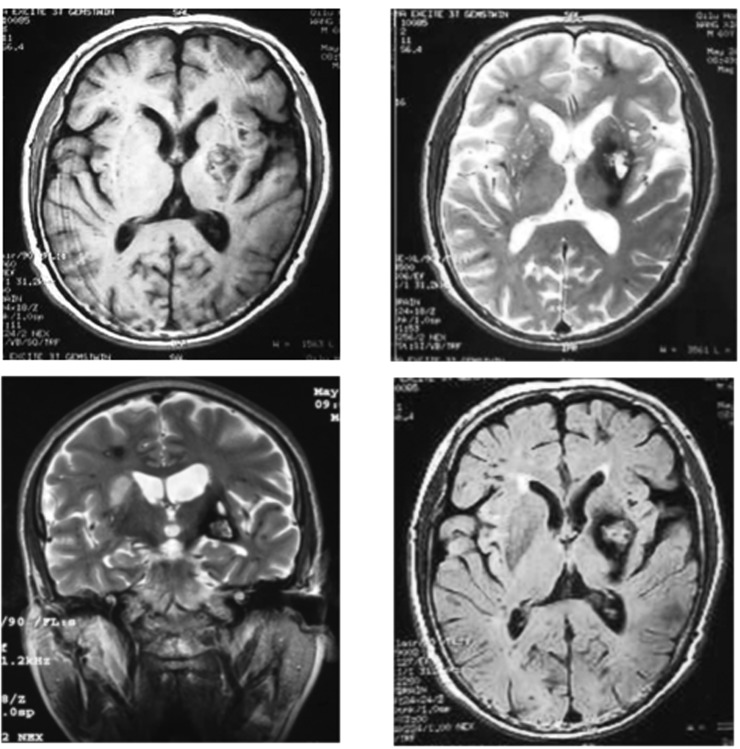
(A, B and C) Foci of CCM show long T_1_ and T_2_ signal intensity in conventional MRI scans and high signal intensity in DWI scans with a distinct boundary. (A and B) The large focus typically has mixed T_1_- signal intensity with a core of mixed high and low T_2_-signal intensity surrounded by a low signal intensity, and a distinct boundary. (D) These lesions demonstrated high signal intensities surrounded by a low signal intensity on T_2_Flair images. CCM, cerebral cavernous malformation; MRI, magnetic resonance imaging; DWI, diffusion-weighted imaging.

Only 3 patients had larger vascular malformations visible in CT scans. The foci were irregular hypodense and hyperdense on the CT scans, usually complicated with hemorrhage and calcification, and were surrounded by a distinct boundary without edema or occupied effect (Fig. 1). With T_1_WI, T_2_WI, T_2_Flair and DWI sequences, the foci showed long T_1_ and long T_2_ signal intensities with distinct boundaries. These lesions demonstrated high signal intensities on T_2_Flair images, high and low mixed signals on T_1_WI scans, and a core of mixed signal intensity with a surrounding rim of decreased signal intensity on T_2_WI scans with a distinct boundary and no evident edema. A low signal black rim that completely surrounded the larger lesion with a mixed signal intensity core of high and low signal intensity was typical of CCM; the foci presented as clump-like or ‘popcorn-like’ with a distinct boundary surrounding the foci without edema or occupied effect (Fig. 2).

### Appearance of CCM by brain MRA

**Figure 3.**
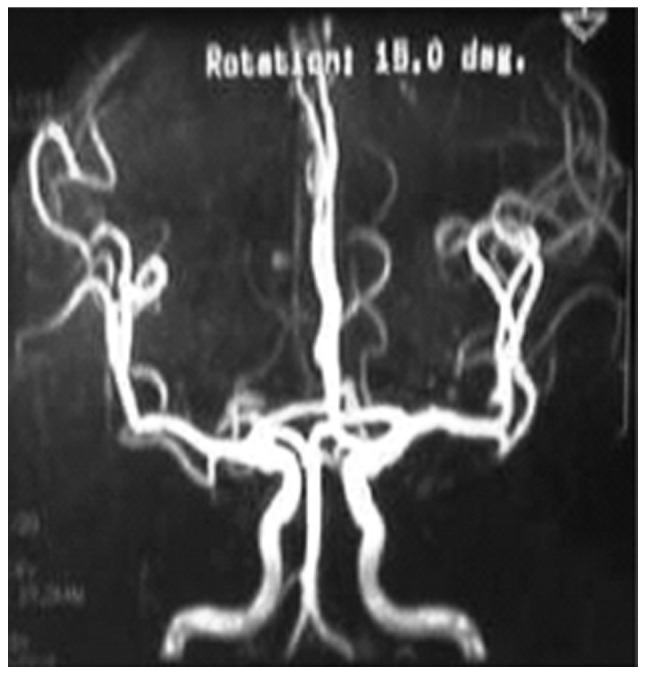
Cerebral vessels by brain MRA showed cerebral atherosclerosis and minor angiostenosis. MRA, magnetic resonance angiography.

All patients underwent MRA and revealed no abnormality, with the exception of 2 elderly patients who suffered cerebral atherosclerosis and minor angiostenosis (Fig. 3).

### Appearance of CCM in SE and GRE T_2_^*^-WI scans

**Figure 4.**
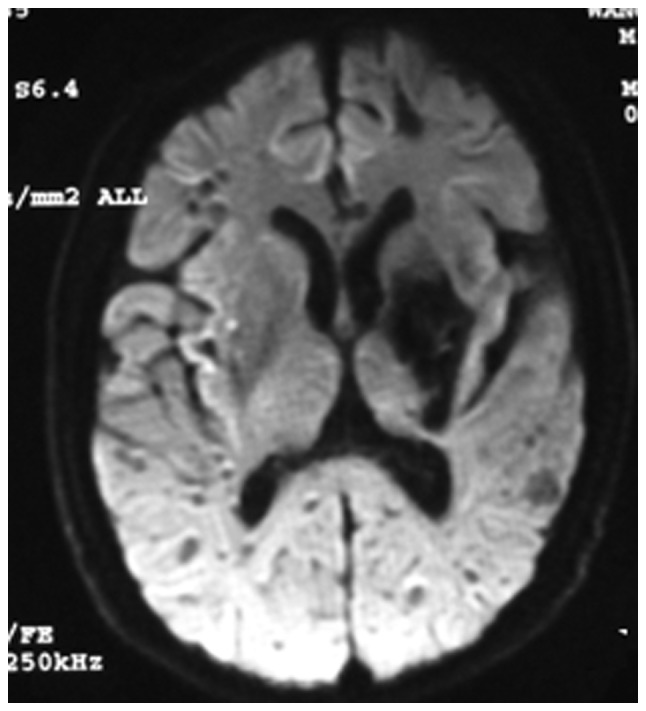
SE scan showed foci of mixed high and low signal intensity surrounded by a rim of low signal intensity but the foci were not distinct. SE, spin-echo.

**Figure 5.**
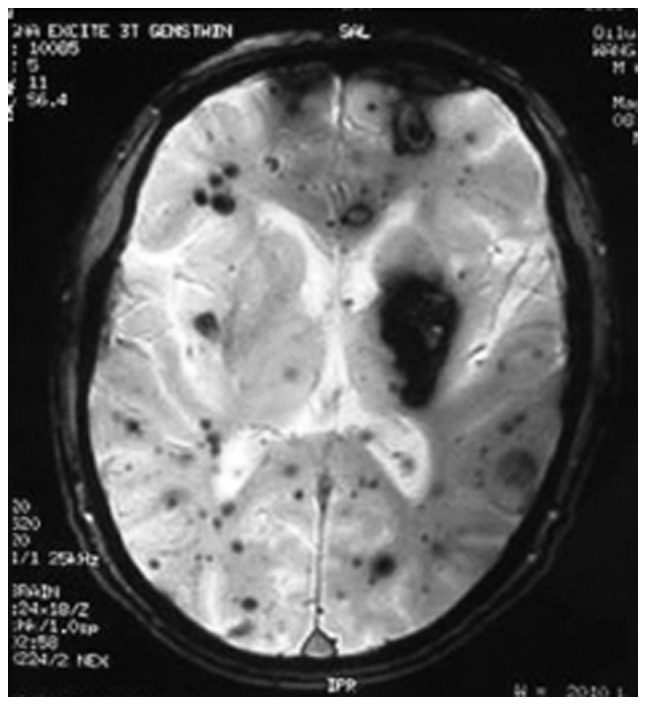
Foci of various sizes were distinctly shown by GRE T_2_^*^-WI. The typical focus was characterized by a mixed high and low signal intensity surrounded by a black rim of low signal intensity. GRE T2*-WI, T2*-weighted gradient echo imaging.

The foci were characterized by specific mixed high- and low-signal intensities surrounded by a black rim of decreased signal intensity with a distinct boundary and no occupied effect. Foci at various locations and of different sizes were seen clearly by GRE T_2_^*^-WI and more distinctly than by SE (Figs. 4 and 5).

### Different appearance of CCM in CT, conventional MRI and GRE T_2_^*^-WI scans

As shown in Table I, 12 of the 26 members of the 2 families were diagnosed with CCM by GRE T_2_^*^-WI. The patients were examined by brain CT and conventional MRI prior to GRE T_2_^*^-WI. Multiple foci were detected in all CCM patients by GRE T_2_^*^-WI. The numbers of foci observed ranged from 8 to 85 (mean 23) and the foci ranged in size from 2 to 55 mm. Abnormal signal intensities were observed in the images of 9 patients (9/12) by brain T_1_WI, 8 patients (8/12) by T_2_WI, 9 patients (9/12) by T_2_Flair and 10 patients (10/12) by DWI, and 3, 3, 4 and 7 patients were diagnosed with CCM, respectively. The mean numbers of foci detected were 5 (3–26) using T_1_WI, 5 (2–24) using T_2_WI, 6 (3–29) using T_2_Flair and 7 using DWI (6–35). However, of the patients subjected to SE and GRE T_2_^*^-WI, 11 patients (11/12) and 12 patients (12/12), respectively, had abnormal signal intensities, and 9 patients (9/12) and 12 patients (12/12), respectively, were diagnosed with CCM. The sensitivity of GRE T_2_^*^-WI was much higher than that of CT (P<0.05), but the difference between GRE T_2_^*^-WI and other conventional MR sequences had no statistical significance. The mean numbers of detected foci were 17 (7–45) when SE was used and 23 (8–85) when GRE T_2_^*^-WI was used. Three patients were observed to have abnormal foci by CT and all were diagnosed with CCM. Three very small CCM lesions were easily detected with GRE T_2_^*^-WI but not diagnosed with SE. In addition, multiple foci were detected with greater sensitivity by GRE T_2_^*^-WI than by SE and other MR sequences (P<0.05).

### Distribution of foci by GRE T_2_^*^-WI (mean)

The foci were distributed as follows: cortex-subcortex, 4 (2–22); basal ganglia, 10 (3–36); thalamencephalon, 4 (1–8); cerebellum, 3 (2–11); and brainstem, 2 (1–7).

## Discussion

CCM is a common cerebral malformation and classified as familial (50–67%) and sporadic (33–50%) forms based on patterns of onset (5–10). Sporadic cases are frequently reported while familial CCM is rare in China. CCM is composed of numerous micrangium with dilated thin walls separated by nerve fibers. However, normal brain tissue is not observed among blood sinuses that lack elastic fibers and muscle tissues. Hemorrhage is common. Progressive neurological deficits, seizures and headaches are reported to be characteristic of episodes of rebleeding in the brainstem and cerebral cavernomas (11–13). The prevalence of CCM in the general population has been estimated to be 0.4–0.8%, accounting for 10–20% of vascular malformations in the central nervous system (10,14–16). CCM was previously diagnosed *post mortem* but may be diagnosed *ante mortem* with the widespread use of CT and MR techniques. With brain MRI in particular, cases of FCCM and multiple brain CCM may be identified and the natural history and developmental process of CCM are well understood (9,17). The features of FCCM on brain CT and MR images are associated with the pathological structure and the development of cavernous vascular malformations (18). Only 3 of the12 patients suffering from clinical symptoms were diagnosed with CCM by brain CT, a positive diagnosis rate of 25%. The 3 patients presented with headache, movement and sensory problems affecting the limbs or speech disfluency. Foci having an abnormally high density or mixed high and low density were observed by brain CT; calcification and a distinct boundary without a surrounding zone of edema or occupied effect distinguished them from cerebral hemorrhage. The foci were located in the subcortex or basal ganglia region. The 3 patients were finally diagnosed as having multiple CCM of the brain by MRI which revealed numerous microfoci in addition to the larger foci identified by CT. The microfoci were not shown in the brain CT images. Similarly, the 9 other patients with brain multiple vascular malformations ascertained by MRI had no or minor symptoms or signs due to the microfoci which were not shown by CT. The foci identified in the brainstem and cerebellum by MRI were also not shown by CT. Therefore, brain CT is only sensitive to the larger vascular malformations with hemorrhage and calcification. The foci which showed irregular mixed high and low density without specificity and microvascular malformations were not shown by CT. The diagnosis of FCCM by brain CT is clearly limited.

Brain MRI is much more sensitive to CCM than CT, particularly when the focus is located in the posterior cranial fossa or brainstem (19,20). The focus of CCM, particularly of CCM with chronic repeated hemorrhage, is clearly seen by MRI due to methemoglobin, hemosiderin deposition, thrombus, calcification and surrounding reactive gliosis induced by repeated and multiple hemorrhaging of the vascular malformation. The MRI signal intensities of the foci vary according to the hemorrhagic period. The foci show high T_1_-signal intensity and low T_2_-signal intensity in the acute stage, and mixed high and low signal intensity in chronic phase due to nomadic diluted methemoglobin and hemosiderin deposition. The phenomenon of hemosiderin deposition occurs 1 week after hemorrhage of the CCM and shows first at the perimeter and extends to the core of focus. The area of hemosiderin deposition shows lasting low T_1_ and T_2_ signal intensity, clearly by T_2_WI with a surrounding black rim of hemosiderin deposition enhanced by reactive gliosis which shows long T_1_ and T_2_ signal intensity. However, it was not possible to identify all CCM patients and their respective foci using conventional MRI. T_1_WI, T_2_WI, T_2_Flair and DWI identified abnormal signal intensities in 9, 8, 9 and 10 of the 12 patients, respectively, and 3, 3, 4 and 7 of the patients, respectively, were diagnosed with CCM. The mean numbers of foci detected were 5, 5, 6 and 7, respectively. However, SE and GRE T_2_^*^-WI detected abnormal signal intensities in 11 and 12 patients, respectively, and diagnosed CCM in 9 and 12 patients, respectively; the mean numbers of foci detected were 17 and 23, respectively. Microvascular malformations from three patients that were not observed by SE were easily identified using GRE T_2_^*^-WI. GRE T_2_^*^-WI was more sensitive to multiple foci than SE, and accurately and reliably ascertained CCM of the brainstem and cerebellum. Conventional MRI has more notably increased the detection of abnormal foci than CT, but has a limitation in decision of focal property, namely, although the focus was observed the etiological diagnosis of the focus was confined. We suggest that the reason for the difference between GRE T_2_^*^-WI and other conventional MR sequences being not statistically significant is that the number of patients studied is too few. However, GRE T_2_^*^-WI not only accurately identified all foci of each size but also provided a qualitative diagnosis based on their features. Therefore, we consider that GRE T_2_^*^-WI is a ‘gold’ standard for the diagnosis of CCM and is almost as reliable as autopsy.

The 12 patients with FCCM all had multiple foci; the foci ranged in number from 8 to 85 and in size from 2 to 55 mm. The 3 patients who had larger foci with repeated hemorrhage and calcification were clearly identified with CT. Multiple foci were a common feature of the FCCM cases studied. Non-familial CCM is common in the clinic and usually comprises a single focus. The foci of FCCM may be located in every region of the central nervous system. The foci of our studied patients were almost all supratentorial, while others were subtentorial. The majority were located at the basal ganglia, with a mean of 11 foci, and the larger foci of the 3 patients identified by CT were all located on this region. The next most common locations was the cortex-subcortex with a mean of 4 foci. Foci were also located in the cerebral ganglion (4), cerebellum (3) and brainstem (2). Brainstem CCM foci were mostly located in the pons, and were also located in the midbrain but rarely in the medulla oblongata. Spinal cord MRI was not performed due to the lack of symptoms relating to the spinal cord. Therefore, the current study is not completely representative of the distribution of CCM in the central nervous system in the population.

In conclusion, GRE T_2_^*^-WI is an available technique for detecting FCCM. Conventional brain MRI should be performed to detect multiple foci of CCM, particularly in the members of a family affected by FCCM. In addition, GRE T_2_^*^-WI is significant in the early diagnosis and treatment of FCCM.

## Figures and Tables

**Table I.** Comparison of 12 CCM diagnoses obtained by brain CT, conventional MRI, SE and GRE T_2_^*^-WI.

Variables	T_1_WI	T_2_WI	T_2_Flair	DWI	SE	GRE T_2_^*^-WI	CT
Patients with detected foci	9	8	9	10	11	12	3
Patients with diagnosed CCM	3	3	4	7	9	12	3
Foci of CCM^a^	5 (3–26)	5 (2–24)	6 (3–29)	7 (6–35)	17 (7–45)	23 (8–85)	1 (0–1)

a Mean (range). CCM, cerebral cavernous malformation; CT, computed tomography; MRI, magnetic resonance imaging; SE, spin-echo imaging; GRE T_2_^*^-WI, T_2_^*^-weighted gradient echo imaging; T_1_WI, T_1_-weighted imaging; T_2_WI, T_2_-weighted imaging; T_2_Flair, T_2_-fluid-attenuated inversion recovery; DWI, diffusion weighted imaging.

## References

[1] Hsu FPK, Rigamonti D, Huhn SL, Awad IA, Barrow DL (1993). Epidemiology of cavernous malformations. Cavernous Malformations.

[2] Ohue S, Fukushima T, Friedman AH (2010). Retrosigmoid suprafloccular transhorizontal fissure approach for resection of brainstem cavernous malformation. Neurosurgery.

[3] Labauge P, Brunereau L, Lévy C (2000). The natural history of familial cerebral cavernomas: a retrospective MRI study of 40 patients. Neuroradiology.

[4] Wurm G, Fellner FA (2004). Implementation of T_2_^*^-weighted MR for multimodal image guidance in cerebral cavernomas. Neuroimage.

[5] Denier C, Goutagny S, Labauge P (2004). Mutations within the MGC4607 gene cause cerebral cavernous malformations. Am J Hum Genet.

[6] Kim DS, Park YG, Choi JU (1997). An analysis of the natural history of cavernous malformations. Surg Neurol.

[7] Moriarity JL, Wetzel M, Clatterbuck RE (1999). The natural history of cavernous malformations: a prospective study of 68 patients. Neurosurgery.

[8] Otten P, Pizzolato GP, Rilliet B, Berney J (1989). 131 cases of cavernous angioma (cavernomas) of the CNS, discovered by retrospective analysis of 24,535 autopsies. Neurochirurgie.

[9] Rigamonti D, Hadley MN, Drayer BP (1988). Cerebral cavernous malformations. Incidence and familial occurrence. N Engl J Med.

[10] Robinson JR, Awad IA, Little JR (1991). Natural history of the cavernous angioma. J Neurosurg.

[11] Fritschi JA, Reulen HJ, Spetzler RF, Zabramski JM (1994). Cavernous malformations of the brain stem: a review of 139 cases. Acta Neurochir.

[12] Kondziolka D, Lunsford LD, Kestle JR (1995). The natural history of cerebral cavernous malformations. J Neurosurg.

[13] Krraemer DL, Awad IA (1994). Vascular malformations and epilepsy: clinical considerations and basic mechanisms. Epilepsia.

[14] Gunel M, Awad IA, Finberg K (1996). A founder mutation as a cause of cerebral cavernous malformation in Hispanic Americans. N Engl J Med.

[15] Laberge-le Couteulx S, Jung HH, Labauge P (1999). Truncating mutations in CCM1, encoding KRIT1, cause hereditary cavernous angiomas. Nat Genet.

[16] Del Curling O, Kelly DL, Elster AD, Craven TE (1991). An analysis of the natural history of cavernous angiomas. J Neurosurg.

[17] Moriarity JL, Clatterbuck RE, Rigamonti D (1999). The natural history of cavernous malformations. Neurosurg Clin N Am.

[18] Kattapong VJ, Hart BL, Davis LE (1995). Familial cerebral cavernous angiomas: clinical and radiologic studies. Neurology.

[19] Chi LY, Wang SH, Liu XW (2008). Familial cerebral cavernous malformation: features of clinical manifestation, pathology and imaging in a Chinese family. Cerebrovasc Dis.

[20] Zabramski JM, Wascher TM, Spetzler RF (1994). The natural history of familial cerebral cavernomas: results of an ongoing study. J Neurosurg.

